# RETRACTION: Pyoderma Gangrenosum at Multiple Sites in a Post‐Colostomy Ulcerative Colitis Patient With Chronic Hepatitis B Virus: A Case Report

**DOI:** 10.1111/iwj.70644

**Published:** 2025-04-23

## Abstract

**RETRACTION:** WangB. , LiuT. , LiuF. , and HeY.‐L. , “Pyoderma Gangrenosum at Multiple Sites in a Post‐Colostomy Ulcerative Colitis Patient With Chronic Hepatitis B Virus: A Case Report,” International Wound Journal 17, no. 1 (2020):187–190, 10.1111/iwj.13255.31663272 PMC7948590

The above article, published online on 29 October 2019, in Wiley Online Library (http://onlinelibrary.wiley.com/), has been retracted by agreement between the journal Editor in Chief, Professor Keith Harding; and John Wiley & Sons Ltd. Following an investigation by the publisher, all parties have concluded that this article was accepted solely on the basis of a compromised peer review process. The editors have therefore decided to retract the article. The authors did not respond to our notice regarding the retraction.

**RETRACTION:** B. Wang , T. Liu , F. Liu , and Y.‐L. He , “Pyoderma Gangrenosum at Multiple Sites in a Post‐Colostomy Ulcerative Colitis Patient With Chronic Hepatitis B Virus: A Case Report,” International Wound Journal 17, no. 1 (2020):187–190, 10.1111/iwj.13255.31663272 PMC7948590

The above article, published online on 29 October 2019, in Wiley Online Library (http://onlinelibrary.wiley.com/), has been retracted by agreement between the journal Editor in Chief, Professor Keith Harding; and John Wiley & Sons Ltd. Following an investigation by the publisher, all parties have concluded that this article was accepted solely on the basis of a compromised peer review process. The editors have therefore decided to retract the article. The authors did not respond to our notice regarding the retraction.

